# Supplementary material for: The Oxidative Stability of Champagne Base Wines Aged on Lees in Barrels: A 2-Year Study
Source: Antioxidants (Basel). 2024 Mar 18;13(3):364. doi: 10.3390/antiox13030364 (PMC10968283; doi:10.3390/antiox13030364)
Supplement: Supplementary file 1 [file antioxidants-13-00364-s001.zip › antioxidants-2897965-supplementary.pdf]

antioxidants

Supplementary Materials

Table S1. Summary of the wine samples: Chardonnay musts from two consecutive vintages 2020 and 2021 and two different geographical origins aged in new barrels. Barrels were all provided by the Tonnellerie de Champagne with the same process (double light toasting).

Vintage	2020			2021		
Age of barrels	New barrels			New barrels		
Musts and origins	Chardonnay 2020<br>from Montgueux (Aube)			Chardonnay 2021<br>from Montgueux (Aube)		
Yeasting	IOC 18-2007			Zymaflore® Spark		
Barrels	12 x 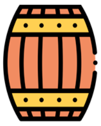	12 x 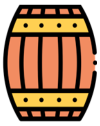	12 x 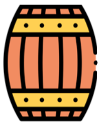	10 x 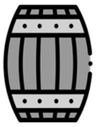	10 x 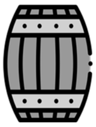	10 x 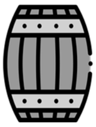
Time ageing and sampling	3 months	6 months	9 months	3 months	6 months	9 months
Barrel specifications	Tonnellerie de Champagne, Hermonville, France<br>Double light toasting (160 °C)					

Table S2. Oenological parameters of musts samples of vintages 2020 and 2021

	Chardonnay musts	
Age of barrels	New barrels	
**Oenological parameter**	Vintage 2020	Vintage 2021
Gluconic acid (g/L)	0.000	0.027
Ammoniacal nitrogen (mg/L)	127	135
Density (g/dm^3) at 20 °C	1077	1071
Potential alcohol (% v/v)	10.7	10.0
Total SO_2 (mg/L)	57	55
pH	3.11	3.05
Total acidity (g H_2SO_4/L)	6.6	8.9
Volatile acidity (g/L acetic acid)	-	0.04
Turbidity (NTU)	39	19

Table S3. Oenological parameters of wines samples fermented and aged in new barrels of vintages 2020 and 2021

	Chardonnay wines						
	New barrels						
	Vintage 2020*				Vintage 2021		
**Oenological parameter**	End of AF	3 months	6 months	9 months	3 months	6 months	9 months
Alcohol strength (% v/v)	-	11.2 ± 0.1	11.3 ± 0.1	11.4 ± 0.1	11.0 ± 0.0	11.1 ± 0.0	11.1 ± 0.0
pH	3.08 ± 0.01	2.99 ± 0.01	3.07 ± 0.01	3.12 ± 0.02	2.89 ± 0.01	2.93 ± 0.01	2.95 ± 0.00
Total acidity (g H_2SO_4/L)	6.5 ± 0.0	5.9 ± 0.1	5.1 ± 0.2	5.0 ± 0.1	8.3 ± 0.0	8.3 ± 0.0	8.0 ± 0.0
Volatile acidity (g/L acetic acid)	0.20 ± 0.01	0.28 ± 0.01	0.28 ± 0.02	0.39 ± 0.03	0.47 ± 0.01	0.47 ± 0.03	0.59 ± 0.04
Total SO_2 (mg/L)	44 ± 1	55 ± 1	60 ± 2	56 ± 6	63 ± 2	62 ± 2	62 ± 3
Free SO_2 (mg/L)	13 ± 1	13 ± 1	15 ± 2	15 ± 2	20 ± 1	14 ± 1	15 ± 0
Residuals sugars (g/L)	1.6 ± 1.7	0.4 ± 0.1	0.4 ± 0.1	0.4 ± 0.1	0.4 ± 0.1	0.4 ± 0.1	0.4 ± 0.0

*wines from vintage 2020 underwent MLF.

Table S4. Raw data of Ec_{20} and molecular ellagitannins concentrations of each barrel for vintages 2020 and 2021

		Ec_{20}			Castalin (mg equivalent ellagic acid/L)			Castalagin (mg equivalent ellagic acid/L)			Vescalagin (mg equivalent ellagic acid/L)		
	Barrel	T3	T6	T9	T3	T6	T9	T3	T6	T9	T3	T6	T9
Vintage 2020 (12 new barrels)	B23	23	23	16	0.8	0.8	1.3	4.0	5.1	6.7	3.9	4.6	5.7
	B24	23	23	17	0.7	1.3	1.6	2.6	5.3	7.1	2.5	4.5	6.0
	B25	23	23	16	0.7	1.2	1.5	3.5	6.1	8.7	2.8	4.8	6.6
	B26	23	23	17	0.4	1.2	1.4	2.9	5.9	7.5	2.3	4.3	5.3
	B27	23	23	17	0.4	1.3	1.5	3.1	6.2	7.1	2.8	5.0	5.8
	B28	22	21	16	0.8	1.5	1.3	4.2	8.1	8.6	3.7	6.8	7.0
	B29	22	21	14	1.0	1.9	1.5	6.3	9.2	13.0	4.6	6.4	8.7
	B30	23	20	15	0.7	1.3	1.5	4.6	8.7	10.6	4.0	7.1	8.4
	B31	22	18	14	0.7	1.3	1.8	6.8	10.8	13.0	5.9	8.6	10.5
	B32	21	20	13	0.9	2.0	1.7	5.9	11.3	14.3	5.8	9.9	12.7
	B33	23	20	15	0.9	1.5	1.4	4.3	9.2	9.6	4.0	7.3	7.3
	B34	22	18	14	0.9	1.6	1.8	5.5	12.0	13.0	4.3	8.6	9.2
Vintage 2021 (10 new barrels)	B50	16	19	17	0.6	0.8	0.9	6.1	9.3	11.2	5.3	7.4	8.6
	B51	16	16	17	0.5	0.8	1.1	7.1	10.1	11.7	5.0	6.3	7.2
	B52	16	22	19	0.6	0.5	1.2	3.7	4.9	6.2	2.8	3.4	4.1
	B53	15	19	17	0.5	0.8	0.9	5.9	8.8	10.9	3.8	5.3	6.2
	B54	16	21	19	0.7	0.6	1.1	3.3	4.8	5.7	2.4	3.1	3.4
	B55	15	19	17	0.9	1.4	1.7	5.4	7.6	9.2	3.4	4.3	4.8
	B56	18	22	19	0.4	0.9	0.9	4.3	5.9	7.3	4.3	5.2	5.9
	B57	16	19	17	0.7	0.5	0.8	6.8	11.0	13.3	5.6	7.7	9.6
	B58	15	18	15	0.7	1.2	1.3	10.3	14.3	18.9	7.7	10.3	12.3
	B59	16	18	17	0.6	0.9	1.1	7.4	11.7	14.5	6.3	8.9	10.4

antioxidants MDPI

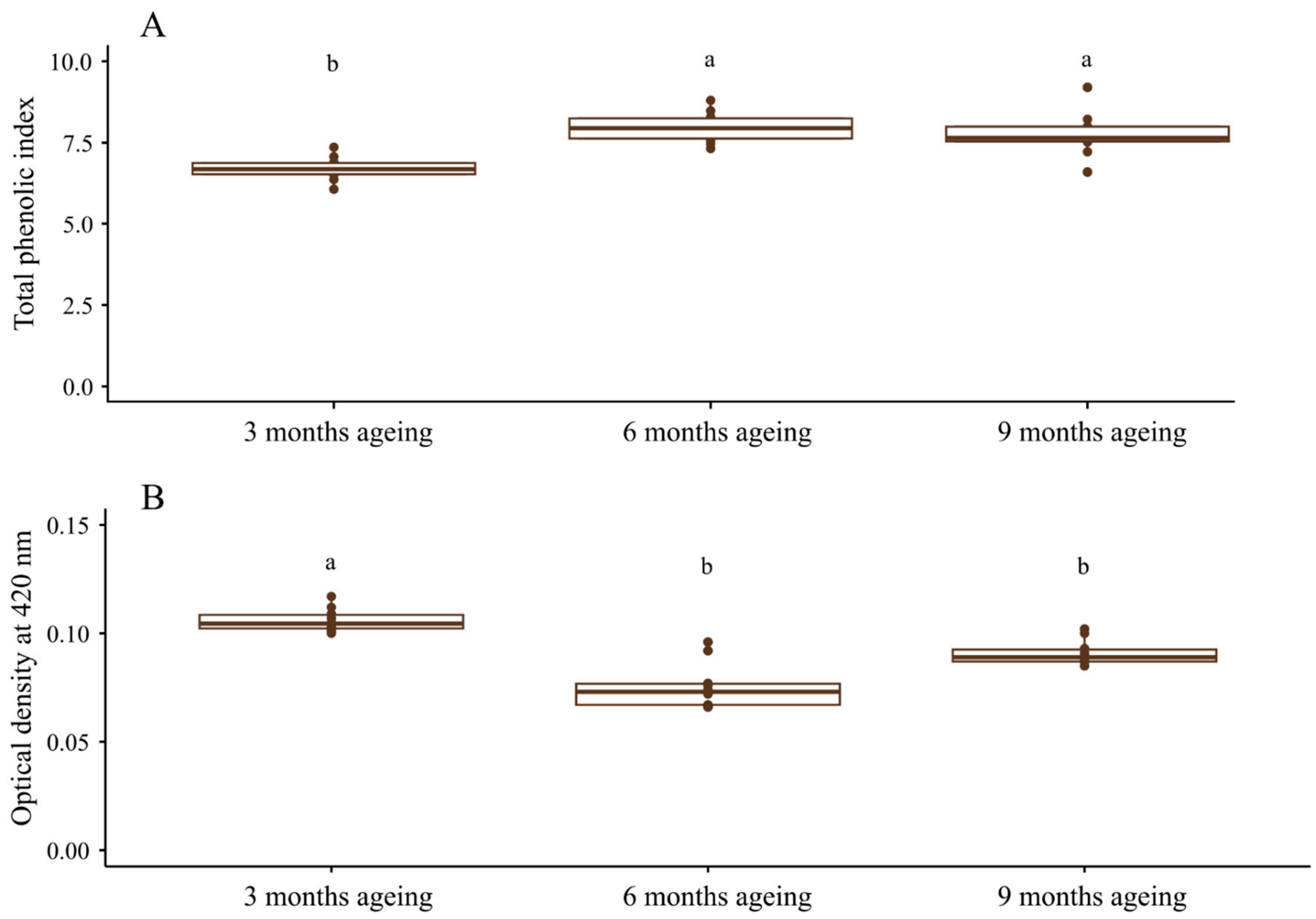

Figure S1. Evolution of I_{280} (A) and A_{420} (B) of 10 wines aged in new barrels during 9 months (Kruskal-Wallis test, *p-value* < 0.05)

antioxidants

MDPI

Table S5. Summary of discussed features with their characteristics. Level of confidence are adapted from Schymanski et al., 2014 [19]

General information		Annotations			Additional details	
RT [min]	Neutral mass [M] [Da]	Molecular Formula	Compound annotation	Annotations' level	Behavior during barrel ageing	AM-RS or AM-NU
3.7	302.0420	$C_{15}H_{10}O_7$	Quercetin	1	UP	strong AM-RS
3.6	138.0316	$C_7H_6O_3$	Salicylic acid	1	UP	weak AM-RS
2.1	154.0265	$C_7H_6O_4$	Gentisic acid	1	UP	strong AM-RS
2.9	302.0057	$C_{14}H_6O_8$	Ellagic acid	1	UP	strong AM-RS
0.9	170.0213	$C_7H_6O_5$	Gallic acid	1	UP	strong AM-RS
1.1	934.0707	$C_{41}H_{26}O_{26}$	Castalagin or vescalagin	3	UP	strong AM-RS
0.9	174.0526	$C_7H_{10}O_5$	Shikimic acid	1	UP	-
2.8	244.1783	$C_{12}H_{24}O_3N_2$	LL	3	UP	-
0.6	180.0630	$C_6H_{12}O_6$	*myo*-inositol	1	UP	AM-RS
1.4	134.0216	$C_4H_6O_5$	Malic acid	2	DOWN	-
0.7	192.0268	$C_6H_8O_7$	Citric acid	2	DOWN	-
1.4	312.0480	$C_{13}H_{12}O_9$	Caftaric acid	1	DOWN	-
1.6	204.0896	$C_{11}H_{12}O_2N_2$	L-tryptophan	1	-	AM-RS
1.9	191.0614	$C_7H_{13}O_3NS$	N-acetyl-L-methionine	2	UP	AM-RS
2.9	112.0158	$C_5H_4O_3$	2-furancarboxylic acid	3	UP	AM-RS
0.9	153.0089	$C_3H_7O_4NS$	L-cysteine sulfinic acid	3	-	AM-NU decreasing with barrel ageing
2.2	307.0826	$C_{10}H_{17}O_6N_3S$	Glutathione	2	-	AM-NU decreasing with barrel ageing
3.4	322.0933	$C_{10}H_{18}O_6N_4S$	CNS or CGGS	3	-	AM-NU decreasing with barrel ageing
1.6	617.1151	$C_{23}H_{27}O_{15}N_3S$	2-S-glutathionyl caftaric acid (GRP)	3	-	AM-NU decreasing with barrel ageing
1.1	922.1800	$C_{33}H_{42}O_{21}N_6S_2$	2,5-di-S-glutathionyl caftaric acid (GRP2)	3		AM-NU decreasing with barrel ageing
3.8	361.2012	$C_{19}H_{27}O_4N_3$	FPV	3	-	AM-NU increasing with barrel ageing
3.1	333.1694	$C_{17}H_{23}O_4N_3$	AFP	3	-	AM-NU increasing with barrel ageing
1.8	154.0261	$C_7H_6O_4$	Gentisic acid (or isomers)	3	-	AM-NU increasing with barrel ageing
4.5	140.0468	$C_7H_8O_3$	Gentisyl alcohol (or isomers)	3	-	AM-NU increasing with barrel ageing

Table S6. Putative annotation of some derivatized compounds contributing to the AM-NU fraction. All detected features have been detected in free form. Putative peptides are amino acids combination and not amino acids sequence. Levels of confidence are adapted from Schymanski et al., 2014; Sumner et al., 2014 [19,20]: [a]1-9, [b]3-4.5. * all KEGG propositions.

General information			KEGG Annotation				Home-made peptides database Annotation				Intra-laboratory white wine nucleophilic compounds list		(1) KEGG Putative Name (2) Home-made peptides Databases (3) Identification
RT [min]	Detected Mass	Neutral Mass [M] [Da]	Identity (ID)	Neutral Mass [Da]	Molecular Formula	Error [ppm]	Peptide Sequences	Neutral Mass[Da]	Molecular Formula	Error [ppm]	Identity	Error [ppm]	
0.7	302.0784	180.0417	C01179 / C01197 / C01405 / C01481 / C05350 / C12623	180.0423	$C_9H_8O_4$	3.3826							*Caffeic acid (1)
0.9	329.1749	207.1382	C09912 / C10567	207.1372	$C_{11}H_{17}ON_3$	-4.7698							Alchornine (1) Arenaine (1)
2.7	342.1206	220.0839	C00643 / C01017 / C09985 / C19716 / C21777	220.0848	$C_{11}H_{12}O_3N_2$	3.9167							*Hydroxy-L-tryptophan (1)
2.8	195.0350	72.9983	C18587	72.9986	C_2H_3NS	4.7946							Methyl isothiocyanate (1)
3.7	330.1094	208.0727	C02381 / C05610 / C09816	208.0736	$C_{11}H_{12}O_4$	4.0322							*Sinapaldehyde (1)
4.5	278.0347	155.9980	C02357 / C02370	155.9978	$C_7H_5O_2Cl$	-1.3013							*2-Chlorobenzoate (1)
0.9	237.0989	115.0622					P	115.0633	$C_5H_9O_2N$	9.8121			
2.4	422.2144	300.1777					GKP	300.1798	$C_{13}H_{24}O_4N_4$	6.7493			
0.9	237.0989	115.0622					P	115.0633	$C_5H_9O_2N$	9.8121			

General information			KEGG Annotation				Home-made peptides database Annotation				Intra-laboratory white wine nucleophilic compounds list		(1) KEGG Putative Name (2) Home-made peptides Databases (3) Identification
RT [min]	Detected Mass	Neutral Mass [M] [Da]	Identity (ID)	Neutral Mass [Da]	Molecular Formula	Error [ppm]	Peptide Sequences	Neutral Mass [Da]	Molecular Formula	Error [ppm]	Identity	Error [ppm]	
2.4	422.2144	300.1777					GKP	300.1798	$C_{13}H_{24}O_4N_4$	6.7493			
2.4	452.2247	330.1880					KPS /N /GGVV /ALQ /GA /GGVV /AAGL /AAAV	330.1903	$C_{14}H_{26}O_5N_4$	6.9082			
3.0	243.0553	121.0186					C	121.0198	$C_3H_7O_2NS$	9.4200			
3.1	549.2780	427.2413					KPPS /N /GGPVV /ALPQ /GA /GGPVV /AAGLP /AAAPV	427.2431	$C_{19}H_{33}O_6N_5$	4.2014			
3.1	521.2971	399.2604					KPR	399.2594	$C_{17}H_{33}O_4N_7$	2.3719			
3.2	423.2350	301.1983					GLL /ALV	301.2002	$C_{14}H_{27}O_4N_3$	6.1654			
0.7	244.0836	122.0469									Nu50	6.3909	$C_6H_6N_2O$ (2)
0.9	259.0500	137.0133									Nu59	9.4109	
1,0	273.0659	151.0292									Nu72	7.9077	$C_4H_9NO_3S$ (2)
1,0	347.0660	225.0293									Nu152	6.8946	
1.2	271.0521	149.0154									Nu68	4.0748	
1.2	443.0980	321.0613									Nu239	3.7986	
1.2	273.0658	151.0291									Nu72	8.2387	$C_4H_9NO_3S$ (2)
1.2	566.0594	444.0227									Nu311	5.0888	
1.5	235.0834	113.0467									Nu36	8.1629	$C_5H_7NO_2$ (2)
2.2	737.1350	615.0983									Nu341	2.5593	
2.6	232.0361	109.9994									Nu30	5.4418	
1.2	273.0658	151.0291									Nu72	8.2387	$C_4H_9NO_3S$ (2)
1.2	273.0658	151.0291									Nu72	8.2387	$C_4H_9NO_3S$ (2)

General information			KEGG Annotation				Home-made peptides database Annotation				Intra-laboratory white wine nucleophilic compounds list		(1) KEGG Putative Name (2) Home-made peptides Databases (3) Identification
RT [min]	Detected Mass	Neutral Mass [M] [Da]	Identity (ID)	Neutral Mass [Da]	Molecular Formula	Error [ppm]	Peptide Sequences	Neutral Mass [Da]	Molecular Formula	Error [ppm]	Identity	Error [ppm]	
1.2	566.0594	444.0227									Nu311	5.0888	
1.5	235.0834	113.0467									Nu36	8.1629	$C_5H_7NO_2$ (2)
2.2	737.1350	615.0983									Nu341	2.5593	
2.6	232.0361	109.9994									Nu30	5.4418	
2.7	325.0607	203.0240									Nu126	4.6478	$C_7H_9NO_4S$ (2)
2.8	345.0867	223.0500									Nu149	5.1833	
3.0	285.0661	163.0294									Nu85	4.4082	$C_5H_9NO_3S$ (2)
3.0	315.0763	193.0396									Nu112	4.8036	
3.1	285.0117	162.9750									Nu84	4.0270	$C_4H_5NO_2S_2$ (2)
3.2	355.0710	233.0343									Nu158	4.7692	$C_8H_{11}NO_5S$ (2)
3.2	327.0761	205.0394									Nu129	5.2584	
3.2	325.0604	203.0237									Nu125	5.3949	$C_7H_9NO_4S$ (2)
3.3	410.0984	288.0617									Nu207	4.9901	$C_{15}H_{12}O_6$ (2)
3.4	232.0361	109.9994									Nu31	5.8514	
3.4	234.0520	112.0153									Nu34	3.0679	$C_5H_4O_3$ (2)
3.5	274.0465	152.0098									Nu74	4.9499	
3.6	290.0776	168.0409									Nu93	4.4766	$C_8H_8O_4$ (2)
3.7	371.1354	249.0987									Nu174	5.1477	
3.8	250.0830	128.0463									Nu55	6.5539	$C_6H_8O_3$ (2)
3.9	165.0244	42.9877									Nu7	3.7282	
4.0	243.0887	121.0520									Nu45	3.6297	C_7H_7NO (2)
4.1	463.0706	341.0339									Nu254	4.1227	
4.2	204.0414	82.0047									Nu16	6.8727	
4.3	195.0345	72.9978									Nu14	8.9286	C_2H_3NS (2)
4.3	223.0658	101.0291									Nu25	6.1314	C_4H_7NS (2)

General information			KEGG Annotation				Home-made peptides database Annotation				Intra-laboratory white wine nucleophilic compounds list		(1) KEGG Putative Name (2) Home-made peptides Databases (3) Identification
RT [min]	Detected Mass	Neutral Mass [M] [Da]	Identity (ID)	Neutral Mass [Da]	Molecular Formula	Error [ppm]	Peptide Sequences	Neutral Mass [Da]	Molecular Formula	Error [ppm]	Identity	Error [ppm]	
4.3	317.0705	195.0338									Nu117	6.8780	
4.3	405.1558	283.1191									Nu205	5.7848	$C_{17}H_{17}NO_3$ (2)
4.5	262.0828	140.0461									Nu64	7.0333	$C_7H_8O_3$ (2)
4.5	316.0867	194.0500									Nu116	6.0822	
4.8	315.0551	193.0184									Nu111	6.3555	
5.2	345.1018	223.0651									Nu151	8.0940	
6.2	420.1122	298.0755									Nu217	6.2418	$C_{16}H_{14}N_2O_2S$ (2)
6.6	354.0475	232.0108									Nu156	7.4246	
3.1	494.2359	372.1992	C03326	372.2009	$C_{16}H_{28}O_6N_4$	4.5809	EKP /GLPS /APSV /GPTV	372.2009	$C_{16}H_{28}O_6N_4$	4.5836			(Ac)2-L-Lys-D-Ala-D-Ala (1)
3.8	372.1356	250.0989	C09007	250.0994	$C_{17}H_{14}O_2$	1.7993	TM	250.0987	$C_9H_{18}O_4N_2S$	0.8037			2-(2-phenylethyl)-chromone (1)
0.8	191.0943	69.0576	C02420 / C15668	69.0579	C_4H_7N	4.0401					Nu11	5.1050	*1-Pyrroline (1) C_4H_7N (2)
0.9	275.0457	153.0090	C00606	153.0096	$C_3H_7O_4NS$	3.7776					Nu76	3.9839	3-sulfino-L-alanine (1) $C_3H_7NO_4S$ (2)
4.1	280.0504	158.0137	C03591	158.0135	$C_7H_7O_2Cl$	-1.6644					Nu78	6.8518	5-chloro-3-methylcatechol (1)
1.3	237.0989	115.0622					P	115.0633	$C_5H_9O_2N$	9.7252	Nu37	6.3393	$C_5H_9NO_2$ (2) Proline (3)[a]
2.2	300.0767	178.0400					CG	178.0412	$C_5H_{10}O_3N_2S$	6.8749	Nu99	6.6634	$C_5H_{10}N_2O_3S$ (2) Cys-Gly (3)[b]
2.8	303.1094	181.0727					Y	181.0739	$C_9H_{11}O_3N$	6.5940	Nu104	4.7906	$C_9H_{11}NO_3$ (2)
3.6	417.1554	295.1187					N /GGY /GGY	295.1168	$C_{13}H_{17}O_5N_3$	6.1941	Nu210	6.4021	$C_{18}H_{17}NO_3$ (2)

General information			KEGG Annotation				Home-made peptides database Annotation				Intra-laboratory white wine nucleophilic compounds list		(1) KEGG Putative Name (2) Home-made peptides Databases (3) Identification
RT [min]	Detected Mass	Neutral Mass [M] [Da]	Identity (ID)	Neutral Mass [Da]	Molecular Formula	Error [ppm]	Peptide Sequences	Neutral Mass [Da]	Molecular Formula	Error [ppm]	Identity	Error [ppm]	
2.2	429.1195	307.0828	C00051	307.0838	$C_{10}H_{17}O_6N_3S$	3.3086	ACD /CEG	307.0838	$C_{10}H_{17}O_6N_3S$	3.3151	Nu226	2.9683	Glutathione (GSH) (1) GSH (2,3)[a]
2.9	356.1391	234.1024	C21203	234.1021	$C_{10}H_{19}O_4P$	-1.2559	CL	234.1038	$C_9H_{18}O_3N_2S$	6.0828	Nu161	4.4850	Geranyl phosphate (1) $C_9H_{18}N_2O_3S$ (2) Leu-Cys; Ile-Cys (3)[b]
3.0	457.1497	335.1130	C21162 / C21163	335.1117	$C_{15}H_{17}O_6N_3$	-3.8047	CDV /ADM /EGM	335.1151	$C_{12}H_{21}O_6N_3S$	6.2606	Nu247	5.2011	7-Demethylmitomycin A (1) 6-Demethylmitomycin A (1) Mitomycin B (1) $C_{12}H_{21}N_3O_6S$ / $C_{20}H_{17}NO_4$ (2) Val-Cys-Asp (3)[b]
3.1	455.2061	333.1694	C11774	333.1689	$C_{17}H_{23}O_4N_3$	-1.6628	CLV /ALM / AFP	[333.1722; 333.1689]	$C_{14}H_{27}O_4N_3S$ / $C_{17}H_{23}O_4N_3$	[8.4582; 1.6598]	Nu246	6.5294	Primidolol (1) $C_{14}H_{27}N_3O_4S$ (2) Val-Ile-Cys; Val-Leu-Cys (3)[b]

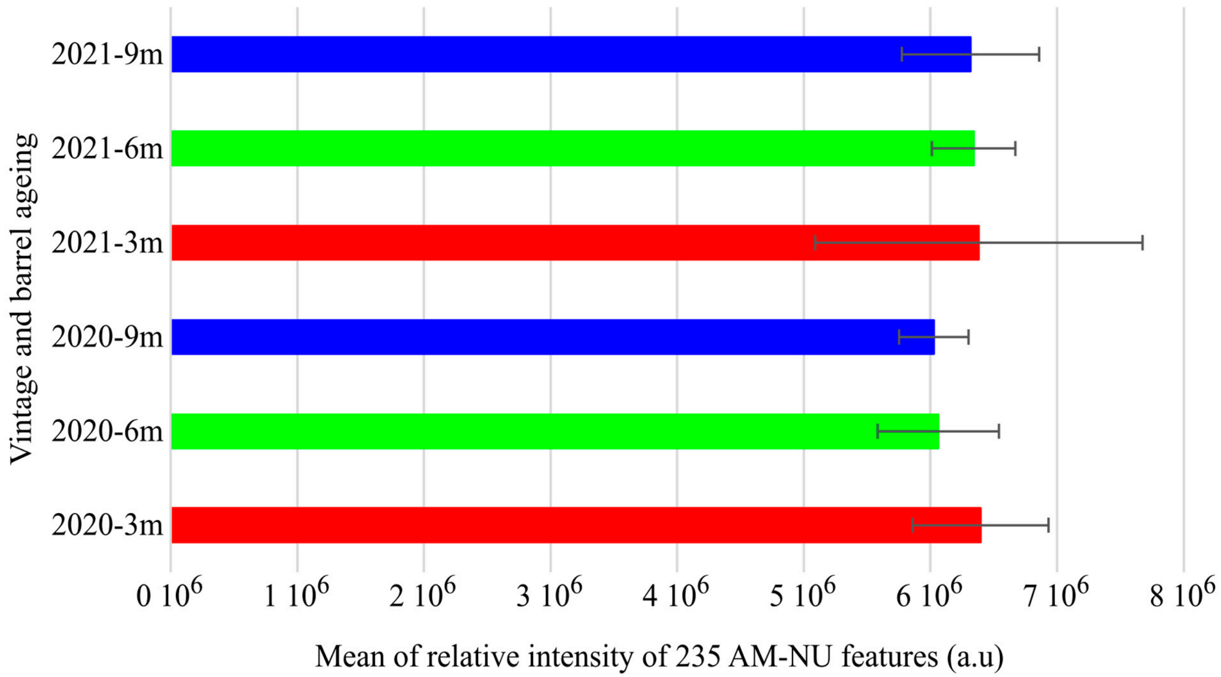

Figure S2. Summary of relative intensities of the assigned 235 AM-NU features found in 66 samples of CH-C-BW from 2020 and 2021 vintages, at 3, 6 and 9 months (m) of barrel ageing in new oak barrels
